# Keystone Flap Reconstruction After Large Mohs Micrographic Surgery Defect of Basal Cell Carcinoma on the Upper Arm: A Case Report

**DOI:** 10.7759/cureus.25709

**Published:** 2022-06-07

**Authors:** Curtis Lockhart, Thomas Knackstedt, Courtney Kromer

**Affiliations:** 1 Department of Dermatology, Case Western Reserve University, School of Medicine and MetroHealth System, Cleveland, USA

**Keywords:** reconstruction, ­skin cancer, basal cell carcinoma, mohs micrographic surgery, keystone flap

## Abstract

Large defects after Mohs micrographic surgery present a reconstructive challenge. The keystone design perforator island flap has proven to be simple and effective in the repair of large skin defects after cancer removal. We present a case of such a defect on the upper arm that was successfully reconstructed with the keystone flap.

## Introduction

Most wounds that appear after skin cancer extirpation by Mohs micrographic surgery can be repaired using linear closure, local tissue advancement, and rotational or transposition flaps. However, there are limited options available for the repair of large defects. Often, skin grafts or secondary intention healing are employed. This is non-ideal for the elderly population, inherently at high risk for complications and better suited for a reliable yet simple reconstructive option for large cutaneous defects. Secondary intention healing may require weeks of wound care and grafts may have a tenuous blood supply and risk of graft failure.

The keystone design perforator island flap, better known as the keystone flap, is a simple yet reliable technique to close large defects after skin cancer removal, especially on the lower extremities [1]. Felix Behan, who developed the keystone flap, reported 99.6% survival among 300 flaps performed, attributing the success of the keystone flap to its robust vascular supply. The use of the keystone flap has been validated in the fields of plastic and dermatological surgery as a reliable reconstructive option with rates of flap failure of less than 1% secondary to the robust vascular supply [2,3]. It is to the surgeon’s advantage to close large Mohs defects using a technique that is versatile, dependable, aesthetically pleasing, and can be done in an outpatient setting without expensive equipment. Most importantly, it is crucial that a reconstructive option provides excellent functional outcomes in the long term. The keystone flap can help achieve these goals, which is illustrated in the following case report.

## Case presentation

**Figure 1:**
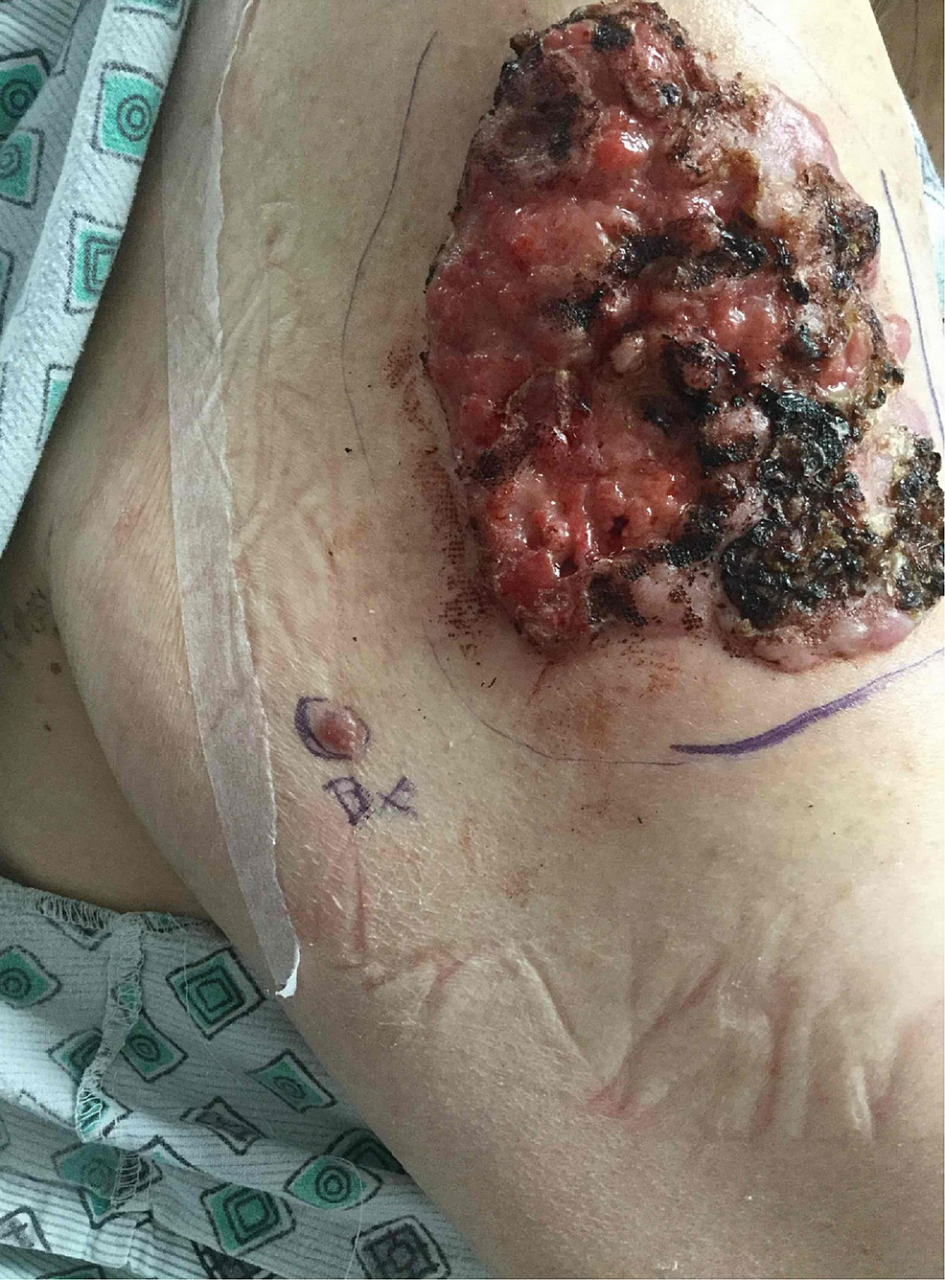
Right upper arm showing large nodular basal cell carcinoma with adjacent basal cell carcinoma

**Figure 2:**
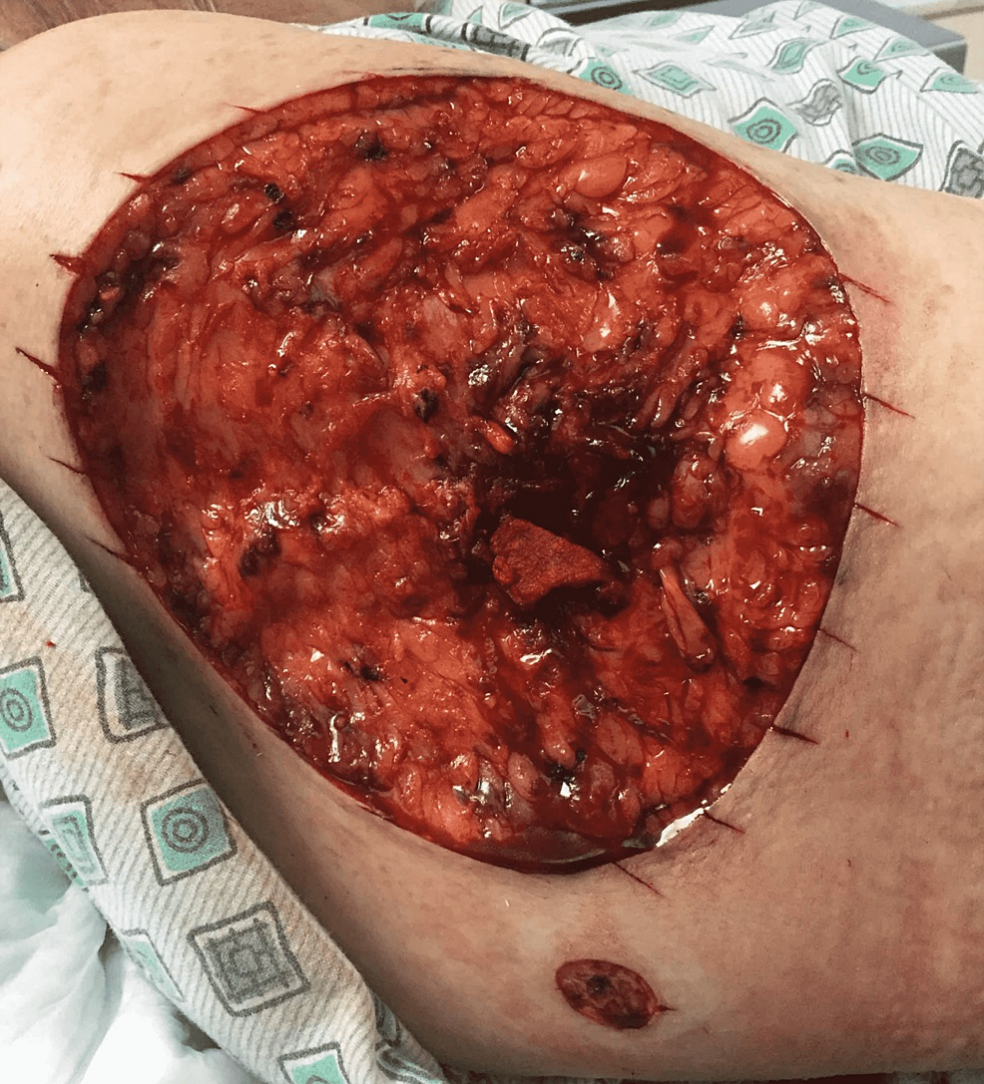
Final Mohs surgical defects measuring 15.5 by 14 cm and 1.8 by 1.5 cm respectively

**Figure 3:**
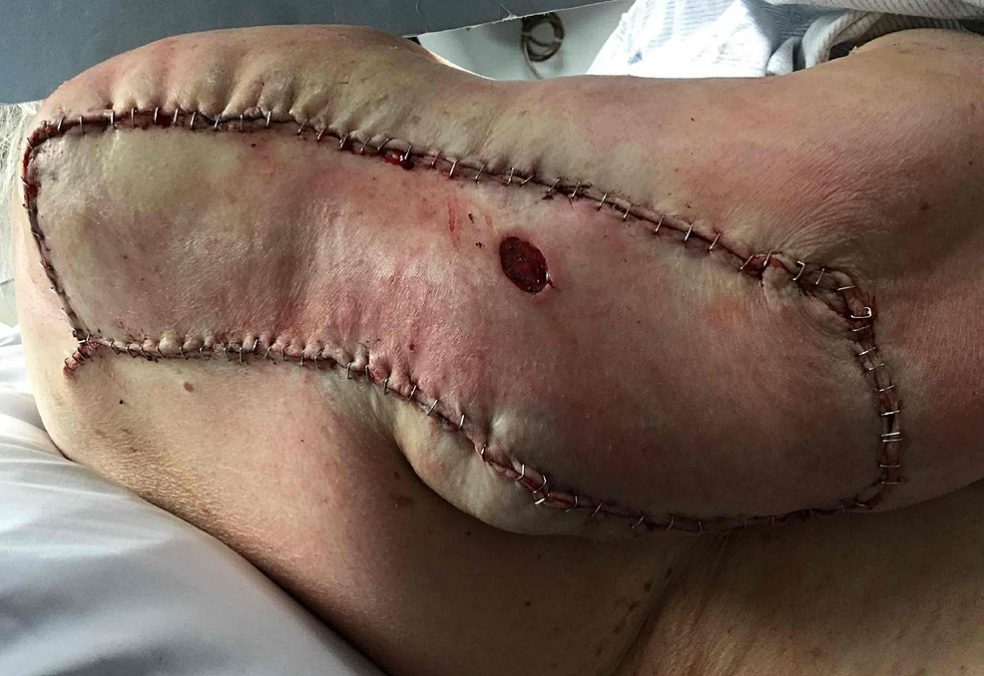
Surgical defect with keystone flap in place. Note the smaller Mohs defect of the adjacent BCC incorporated into the flap BCC: basal cell carcinoma

**Figure 4:**
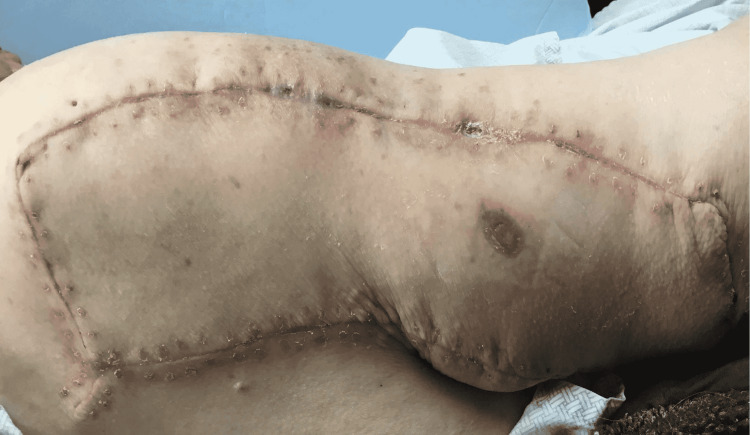
Image at one-month follow-up

A 72-year-old woman with multiple medical comorbidities presented with a neglected 11 by 9-cm nodular basal cell carcinoma on the right upper arm (Figure 1). An additional adjacent nodule was identified at the time of surgery, which was biopsied and found to be consistent with basal cell carcinoma. Both tumors required one stage of Mohs micrographic surgery to achieve tumor-free margins, resulting in 15.5 by 14-cm and 1.8 by 1.5-cm defects, respectively (Figure 2). The optimal skin match was determined to be from the surrounding tissue with a potential tissue reservoir along the posterior arm. An ellipse was drawn along the long axis of the defect. A keystone flap was designed by converting the defect into an ellipse along the long axis and flap borders emanating at 90-degree angles from the apices of the ellipse. The width of the flap was equal to the width of the defect. The incision was taken down to the subcutaneous planes on all sides and the flap was maintained on a central subcutaneous pedicle. Care was taken to preserve central fascial perforators during undermining. Undermining was performed by gentle spreading with sharp scissors rather than sharp cutting to avoid injury to the vasculature. Hemostasis was obtained with pin-point electrocautery. The flap was lifted and advanced anteriorly into the defect on the upper arm. The flap was secured in place with deep 3-0 and 4-0 polyglactin 910 sutures in a buried vertical mattress fashion. The epidermis was reapproximated with staples (Figures 3, 4). The final flap size was 280 cm^2^.

## Discussion

**Figure 5:**
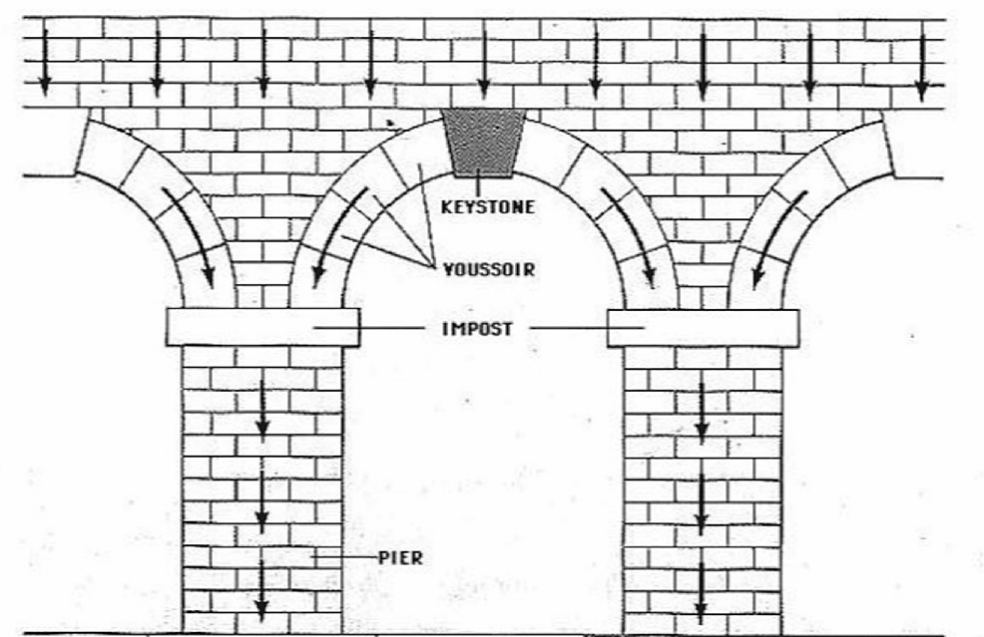
The Roman keystone Picture used free of copyright and royalties from Romanesque Architectural Style of Europe, SlideShare

**Figure 6:**
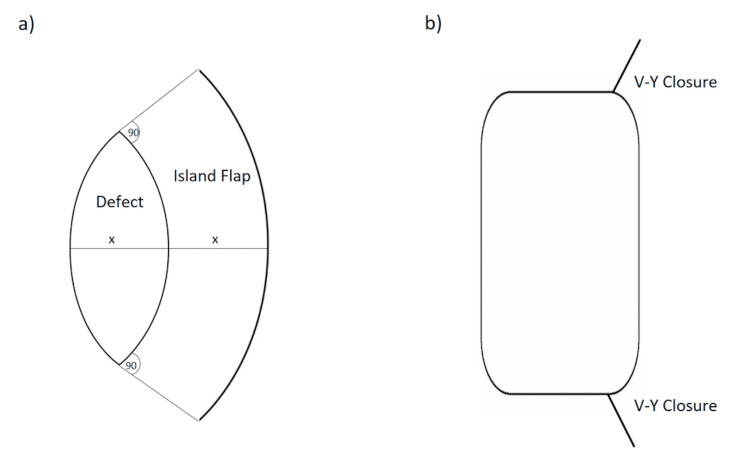
The keystone flap procedure (a) The keystone flap is designed as a trapezoid shape adjacent to the defect with arched borders. The limits of the flap are defined by 90-degree angles from the apices, and flap width is equal to defect width. (b) After the advancement of the flap into the primary defect, closure is accomplished using two V-Y advancement flaps at the apices

The keystone flap's name is derived from the stone used in Roman architecture to bear large structural weight (Figure 5) [1]. This keystone offers structural support by locking the surrounding stones of the arch into place. The keystone flap provides similar stability in the reconstruction of large defects of the skin. The procedure itself is described below and illustrated in Figure 6.

First, the skin should be excised as an ellipse that runs parallel to cutaneous vessels, nerves, and perforating vessels in that location, ideally along the long axis of any preexisting surgical defect. Then, at a 90-degree angle from the apices of the ellipse, an incision is made so that the width of the defect and the flap are 1:1. When choosing which side of the defect to harvest the flap from, it is important to choose the side with greater laxity, as the surrounding tissue will be required to cover the final defect. In this regard, the flap may be slightly undersized if significant secondary tissue movement and laxity from the contralateral defect size are anticipated. While elevating the flap, the deep fascia at the margin of the flap can be preserved or it can be cut through to facilitate greater movement of the flap. Additionally, two keystone flaps can be utilized if the defect is too large. If it is necessary to rotate or move the flap over great distances, the subfascial plane can be dissected. To close the wound, sutures are placed on either side of the flap at points of maximal tension. Then the longitudinal ends are closed as two V-Y advancement flaps, after which redundant tissue can be excised. If the defect cannot be closed in a primary fashion, a skin graft can be employed to cover the remainder of the defect [1]. The procedure is depicted in Figure 6.

An ideal repair after skin cancer removal should be reliable, aesthetically pleasing, allow for efficient and comfortable recovery, and be both time- and cost-effective [1]. The keystone flap accomplishes all of these qualities.

Numerous studies have highlighted the low failure rate and dependability of the keystone flap, with flap failure rates ranging from 0 to 10%, with a significant proportion on the lower end of this spectrum [1,2,3,4,5]. The flap’s extremely low failure rate can likely be attributed to its robust vascular supply via both the superficial and deep perforating vessels, as well as the minimal tension placed on the flap itself [1,6,7]. By not undermining the base, the surgeon preserves its precious vasculature while at the same time allowing the central tissue block to serve as the keystone for the surrounding tissue to lock into place. The bilateral V-Y advancement closure removes excess longitudinal tension from the middle portion of the keystone flap. The skin recruited for the keystone flap also serves as a great color and texture match for surrounding skin as the flap is lifted from adjacent skin.

Multiple studies have underscored the quick recovery time associated with the keystone flap [1,5]. The keystone flap can be performed with minimal equipment in the outpatient Mohs surgery procedure suite with local anesthesia and reasonable operative time. This was especially important for the patient described, who was a poor candidate for general anesthesia and prolonged hospital stay. If the reconstructive area requires excessive local anesthetic due to its large size, lower concentrations (0.5% lidocaine with 1:200,000 epinephrine) or even tumescent anesthesia can be used to avoid toxicity. Additionally, a drain can be placed if there is a concern for hematoma formation, which is kept in place until the drain output is less than 30 mL per day [8]. As this flap is relatively new in the literature, multiple surgeons continue to contribute small modifications to the flap, which have helped to decrease suturing time, improve cosmetic results, and decrease flap donor site size [4,9,10]. Here, we shed light on expanding its utilization beyond the traditional lower extremity location.

The aforementioned strengths of the keystone flap are especially pertinent in the elderly patient population, one that constitutes a large proportion of those who undergo Mohs micrographic surgery. In a population prone to complications, it is invaluable to have simple and reliable reconstructive options. The keystone flap offers just that by minimizing wound checks, reducing postoperative pain, and enabling patients to return to their normal day-to-day lives with good functional results [5]. In a time of medicine that emphasizes patient-directed care, the keystone flap carries significant weight.

## Conclusions

Large defects following Mohs micrographic surgery represent a reconstructive challenge. This is especially critical in the elderly, as reliable repair options can lead to quicker recovery times and functional return for this complication-prone population. The case we described highlights the many benefits of the keystone flap.
